# Evaluation of HIV/AIDS-related mobile health applications content using an evidence-based content rating tool

**DOI:** 10.1186/s12911-021-01498-7

**Published:** 2021-04-24

**Authors:** Ahmad Raeesi, Reza Khajouei, Leila Ahmadian

**Affiliations:** 1 Department of Health Information Sciences, Faculty of Management and Medical Information Sciences, Kerman University of Medical Sciences, Kerman, Iran; 2 Department of Medical Informatics, Faculty of Medicine, Mashhad University of Medical Sciences, Mashhad, Iran; 3 Department of Health Information Sciences, Faculty of Management and Medical Information Sciences, Kerman University of Medical Sciences, Kerman, Iran; 4 HIV/STI Surveillance Research Center, WHO Collaborating Center for HIV Surveillance, Institute for Futures Studies in Health, Kerman University of Medical Sciences, Haft-Bagh Highway, Kerman, Iran

**Keywords:** Content rating, HIV/AIDS, Mobile applications, Evidence-based medicine pyramid, Hierarchy of evidence, EBCRT-mHealth

## Abstract

**Background:**

Despite the increasing number of mobile health applications, the validity of their content is understudied. The objective of this study was to rate the content of HIV/AIDS-related mobile applications and to determine the extent to which evidence-based medicine is being incorporated into their content using a new tool called the Evidence-based content rating tool of mobile health applications (EBCRT-mHealth).

**Methods:**

All available HIV/AIDS-related applications in Iran from Cafe Bazaar and Google Play Store were evaluated. This study was first conducted in 2018, then after almost two years in 2021 was done again. In this study, researchers developed the EBCRT-mHealth tool to rate the content of applications based on the evidence-based medicine pyramid. Its reliability was calculated (α = 0.78), and five specialists confirmed its validity. Two reviewers independently reviewed all HIV/AIDS applications directly downloaded and installed from the Google Play Store and Cafe Bazaar.

**Results:**

Out of 980 retrieved applications, in 2018, 85, and in 2021, 78 applications were included in the study. Only in 17 (28%) out of the 60 in 2018, and 25 (51%) in 2021 Google Play store applications the source of content information was mentioned. All Cafe Bazaar mobile applications mentioned the source of information. The mean rating of all application content in 2018 was 2.38 (SD = 0.74), and in 2021 was 2.90 (SD = 1.35) out of 5. The mean rating of the content of Cafe Bazaar applications in 2018 was 2.10 (SD = 0.49), and in 2021 was 1.94 (SD = 0.29). The mean content rating of Google Play store applications in 2018 was 2.50 (SD = 0.80) and in 2021 was 3.86 (SD = 1.18).

**Conclusion:**

After two years, the rating of the content of HIV/AIDS-related applications available in Iran that existed in Cafe Bazaar decreased from "poor" to "inappropriate". Also, the content score of the Google Play Store applications increased from "poor" to "good". It is critical to ensure the credibility of the sources used in developing their content and removing applications with inappropriate and unreliable content from the App Stores. Also, mobile health application developers should use the highest quality information in their applications.

**Supplementary Information:**

The online version contains supplementary material available at 10.1186/s12911-021-01498-7.

## Background

Providing education to HIV/AIDS patients and others using mobile applications installed on smartphones is one of the most accessible and up-to-date ways which maintains patient confidentiality and reduces social stigma. Mobile applications have many benefits for the management and control of HIV/AIDS including collecting and communicating patient data to care providers, providing educational messages to patients and their families, and meeting the information needs of people to prevent HIV/AIDS [1, 2]. There are 38 million and 61 thousand people living with HIV/AIDS in the world and Iran, respectively in 2019 [3, 4].

Providing information and education to patients and community is one of the most important and effective ways to control HIV/AIDS [5]. In addition to HIV/AIDS prevention, some studies evaluate the application of mobile health in self-care and self-management of patients [6–8]. Moreover, some studies reported on the use of the mobile application in increasing the awareness of the community and care coordination to control HIV/AIDS [9–11]. In the past few decades, evidence-based medicine has played an important role in converting HIV/AIDS from a deadly disease to a chronic disease [12, 13]. Studies [14–16] have shown that people with and at risk of HIV/AIDS are interested in using mobile health (mHealth) applications. Given the impact of mobile application content on the decision making of users and health care providers, and since many people with different levels of knowledge access these apps [17, 18], it is critical to use credible sources and information for the development of the content of mHealth applications [18].

Several tools have been used to evaluate mobile applications so far [19–23]. To our knowledge, none of these tools have been specifically developed to rate the content of applications and to evaluate the extent of scientific evidence used in the content of mHealth applications. Moreover, although some studies [24–27] evaluated HIV/AIDS-related mobile applications, none of these studies have focused on the rating of the content of mobile health applications based on evidence-based medicine. The objective of this study was to rate the content of HIV/AIDS-related mobile applications and to determine the extent to which evidence-based medicine is being incorporated into their content using a new tool called Evidence-based content rating tool of mobile health applications (EBCRT-mHealth).

## Material and methods

This article is part of a larger ongoing study regarding the evaluation of HIV/AIDS-related applications in various terms including their features and content. The purpose of the larger study is to develop tools for the evaluation and rating of the mobile health applications and compare this tools with other developed tools such as mobile apps rating scale (MARS). In the current study only the use of evidence-based medicine in the development of the content of applications is studied. This study was initially conducted from 10 June 2018 to 16 August 2018. After almost two years, due to the non-publication of its results, from 10 January to 5 March 2021, this study is repeated. The study population consisted of all HIV/AIDS-related mobile applications available at Google Play store [28] and Cafe Bazaar [29]. Google Play store is the largest mobile application store in the world [30]. Cafe Bazaar is also the biggest Iranian mobile applications store that has the largest number of Persian mobile applications [31]. We included all HIV/AIDS-related mobile applications in the study. In this study, all included HIV/AIDS mobile applications in the Google Play Store were in English and available around the globe. All applications in the Cafe Bazaar were in Persian and targeted Iranian users or Persian language users, and none of them were bilingual.

Google Play Store and Cafe Bazaar were searched for mobile HIV/AIDS-related applications using the keywords "HIV", "AIDS", "HIV/AIDS", "Human Immunodeficiency Virus", "Acquired Immunodeficiency Syndrome", and their equivalents in Persian. The retrieved applications were installed on the Samsung Galaxy C7000 running Android. If the application was not installed on this smartphone, or there was a difficulty, we checked it on another smartphone (Samsung Galaxy A70). The inclusion criteria for selecting applications were: 1- the possibility of installation on Android OS, 2- in Persian or English languages, 3- available in Iran and 4- the focus of the application on HIV/AIDS.

Two reviewers with health information technology background independently evaluated the mobile applications. These reviewers were trained in the evaluation of health information systems before evaluation. Moreover, prior to the evaluation, both reviewers had received training on how to rate and analyze the content of the applications. After the installation of the applications on smartphones, one of the reviewers evaluated the applications. Afterward, all data stored on the app were removed and the other evaluator performed the evaluation. Whenever there was a difference between the two reviewers' scores, the third evaluator (supervisor) resolved the discrepancy through discussion. All evaluation data were collected on paper forms.

### EBCRT-mHealth tool

**Fig. 1:**
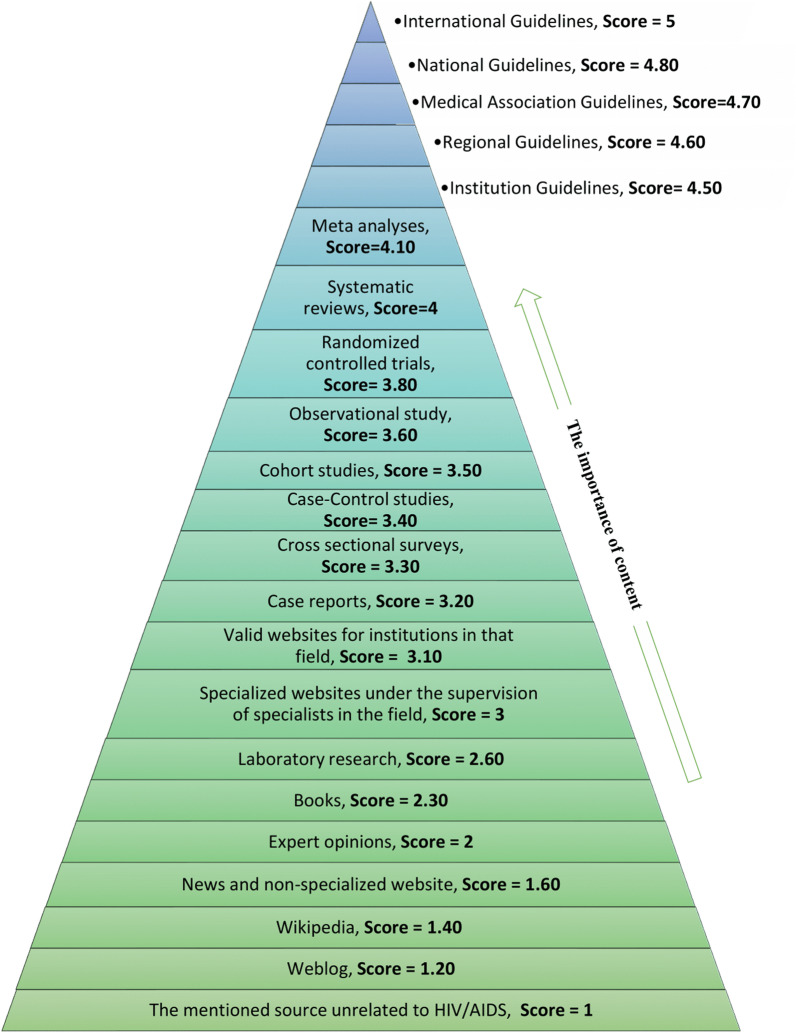
Evidence-based medicine pyramid used in this study

Data were collected using EBCRT-mHealth tool (Evidence-based content rating tool of mobile health applications). This tool was developed by researchers in this study to determine the type of content and rate the information content of HIV/AIDS mobile applications. The EBCRT-mHealth tool is available in Additional file 1. This tool had three sections. The first section included general questions about application specifications. The second section was about the type of sources used to develop different parts of the content based on the hierarchy of evidence pyramid. This part was developed based on the review of previous studies [2, 32–34] concerning the pyramid of evidence. Additionally, we added other sources to the hierarchy of evidence pyramid based on the opinion of two Medical Informatics, two Health Information Management, and one Infectious Disease specialist (Fig. 1).

The third section consisted of 16 questions for rating the applications based on the type of content, including prevention, treatment, education, self-care, medication, symptoms and signs, general information about the illness, health and wellness issues, and other topics. In order to select a content rating method, the rating methods of the previous tools [19–23, 35–37] were first reviewed, and then the content rating method was determined according to the opinion of two medical informatics specialists.

Based on this tool, if a type of content was not available in the application, a zero was assigned by the reviewers. For the available content, a score of 1–5 (inappropriate to excellent) was assigned to the mobile application. If the source of the content was mentioned in an application, that content was considered as evidence-based. The accuracy and validity of the content of applications can be verified if their references and sources are mentioned in the applications.

Figure 1 illustrates how the content of the applications is rated through the evidence-based medicine pyramid. The scoring of the applications content in this study was according to Fig. 1. Based on the evidence-based medicine pyramid, the following rating was devised: a score of 4.5–5 was assigned to guidelines, a score of 3–4.10 to different type of studies from meta-analysis to case studies and specialized websites, a score of 2–2.60 to laboratory research, books, textbooks, and experts opinion, and a score of 1–1.60 to news websites, Wikipedia or blogs and irrelevant sources. The details of these scores for each resource type are stated in Fig. 1. To determine these scores on Fig. 1 we used the division 1–5 (1- inappropriate, 2- poor, 3- acceptable, 4- good, and 5- excellent) and the importance of evidence-based on the hierarchy of evidence. The reviewers also checked whether the content of the applications was consistent with the content of the source used in the development of the applications. To determine the source of information, we considered all items that indicate the source of the content of the application, including links and hyperlinks, the mentioned sources at the end of the content, and the list of sources mentioned separately in the reference section inside the application. If a mobile application's content is based on a specific source, like a national guideline or several national guidelines are available in that mobile application. We assigned the national guideline score (score = 4.8) to that mobile application. To verify an application be a guideline or not to be, we checked the website registered in the Google Play Store, search on the internet, and details inside of that application. If the application used several sources, like a reference list. We calculated the sum of all the sources' scores, and then the average score was considered. No score was assigned to the application if its content did not match the content of a related source. If the source was not available to determine the consistency and accuracy of the content, the application did not receive a score. We used the mean to calculate each applications' average score and the total score of the applications.

The validity of the EBCRT-mHealth tool was confirmed by two Medical Informatics specialists and two Health Information Management specialists, and one Infectious Disease specialist physician. First, an initial draft of the tool is made and then sent to all experts. After applying their comments, it was sent to them again. This cycle continued until all experts approved the validity of the tool. After the validation of this tool, to ensure our developed tool's reliability before using it to all HIV/AIDS applications. We tested our tools on the first 20 Google Play Store applications, which were included in this study based on the inclusion criteria, were evaluated using this tool [19]. Then the reliability of the EBCRT-mHealth was assessed using Cronbach's alpha (α = 0.78). These 20 applications were not excluded from the study. After ensuring the reliability of this tool, we applied it to evaluate other applications. Moreover, to evaluate to what extent mobile app developers respect the confidentiality of their users and their users can use the application secretly, we checked whether information representing HIV/AIDS are used in the title and the logo of each application.

### Statistical analysis method

In this study, descriptive statistics, including mean and standard deviation were used to calculate the application rate. The EBCRT-mHealth score of zero was not used to calculate the mean scores. To rate the mean scores of the EBCRT-mHealth tool, the scores 1–2 was considered as "inappropriate", 2–3 as "poor", 3–4 as "acceptable", 4–5 as "good", and 5 as "excellent". The Kolmogorov–Smirnov normalization test did not confirm the normality of the variables. Therefore, the Spearman correlation test was used to examine the relationship between the EBCRT-mHealth tool score with the number of HIV/AIDS application downloads and the apps rate in the App Stores. Two way mixed inter-correlation coefficient (ICC) was used to calculate internal validity and agreement between reviewers [38]. Descriptive data were analyzed using Microsoft Excel version 2016 and inferential statistics using SPSS version 24.

## Results

**Fig. 2:**
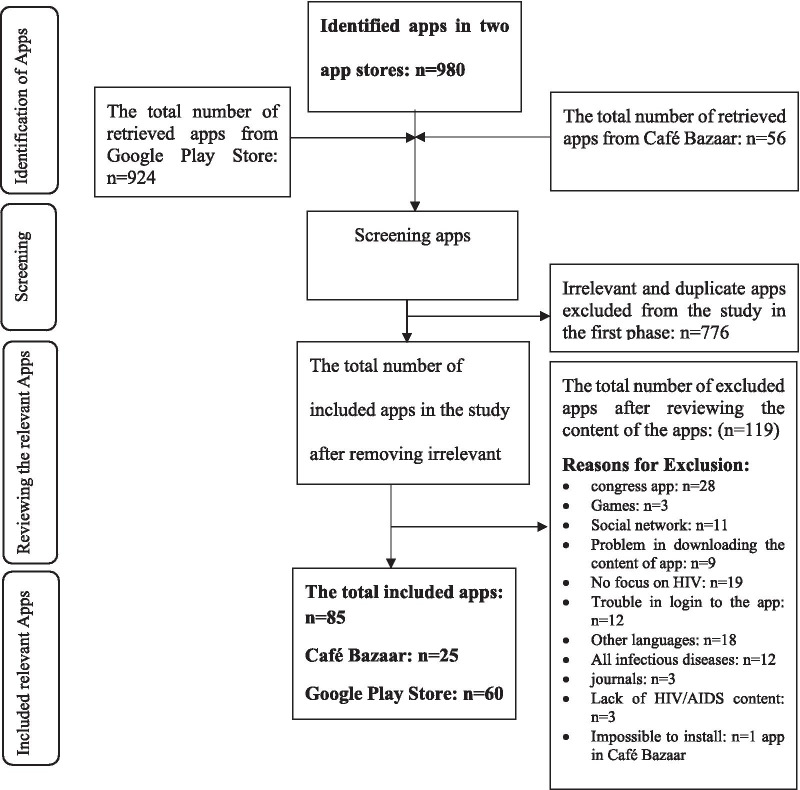
The process of selecting applications for inclusion in the study in 2018

Searching for apps in the Google Play Store and Cafe Bazaar took place on June 10 and 11, 2018, and on 13 and 14, January 2021. In 2018: out of 980 applications, 85 (60 from Google Play store and 25 from Cafe Bazaar) were included in this study. All included google play store applications are free of charge. Four of 25 (16%) Cafe Bazaar applications are paid apps. Figure 2 illustrates the process of selecting applications for inclusion in the study in 2018. A number of applications were excluded due to the issues such as the difficulty of downloading application content (n = 9), inability to login to access the content (n = 12), and inability to install the application (one of the Cafe Bazaar applications).

In 2021, the total number of included mobile applications in both app stores was 78 applications. In Cafe Bazaar, seven new applications have been added, and three applications have been removed from the Cafe Bazaar. The total number of applications included in this study from the Cafe Bazaar were 29 mobile applications. From Google Play Store, 14 applications have been added, and 25 have been removed. The total number of the included applications from the Google Play Store in 2021 was 49 mobile applications.

Descriptive characteristics of included HIV/AIDS mobile applications in 2018 and 2021 are shown in Table 1.

**Table 1:** Descriptive characteristics of included mobile applications in 2018 and 2021

	2018		2021
Mean rating in two app stores	3.86 (SD = 1.63)		3.98 (SD = 1.2)
Median rating in two app stores	4.45 (4.0, 5.0)		4.3 (4.0, 5.0)
Median number of downloads in two app stores	100 (10, 1000)		500 (100, 5000)
Organizational affiliation of HIV/AIDS apps in Google Play store	Unknown	21 (33%) out of 60	20 (41%) out of 49
	Commercial	4 (6%)	3 (7%)
	Governmental	15 (24%)	16 (32%)
	Non-governmental	15 (24%)	5 (10%)
	Academic	8 (13%)	5 (10%)
Organizational affiliation of HIV/AIDS apps in Cafe Bazaar	Unknown	9 (36%) out of 25	15 (52%) out of 29
	Commercial	15 (60%)	12 (42%)
	Non-governmental	1 (4%)	1 (3%)
	Governmental	0	0
	Academic	0	1 (3%)

Nine of the 60 (15%) apps of the Google Play Store, and none of the Cafe Bazaar apps, designed in such way that users can document and collect their personal and health data inside the application. However, our inspection revealed that none of these application allowed user to post their information on social media. Eighteen out of 85 (21%) applications allowed users to share the content of the application (such as: information about HIV/AIDS prevention, etc.) to social media.

Forty-one out of 60 (68%) Google Play Store applications contained the word HIV or AIDS in the title of the applications, and the red ribbon symbol of HIV/AIDS was used in the logo of 29 (48%) applications. Thirty applications (50%) not only contained the HIV/AIDS symbol, but also in their titles, it was clear that the application is related to HIV/AIDS. All Cafe Bazaar applications had these conditions.

In 2018, given that in 17 (28%) of the 60, and in 2021, 25 (51%) out of 49 Google Play store applications, the sources of information were listed, the content of these 17 applications was reviewed and rated. Moreover, all Cafe Bazaar applications (n = 25 and n = 29) in two years were reviewed, as they all had the sources that were used in developing their content.

### Results concerning the review of application content based on the hierarchy of evidence pyramid

In this section, we just reviewed the applications that had mentioned the information acquisition sources. The results of the content type and information sources of Google Play store applications are shown in Table 2. In 2018, of 17 Google Play Store applications, the most commonly content type was “Therapeutic” as same as in 2021. The most commonly used source was “National guideline” in 53% (n = 9) of applications, also in 2021 with 40% (n = 10) out of 25. In 2018 and 2021, among the Google play store applications, none of them reported that they used the evidences of the lower level of the hierarchy of evidences such as Laboratory research, Books, Expert opinions, News and Non-specialized website, Wikipedia, and Weblogs.

**Table 2:** The results of the content type and information sources of Google Play store applications

Content-type	General information on disease		Therapeutic		Prevention		Self-care and self-management		Pharmaceutical information		Signs and symptoms	
	2018 N (F)*	2021 N (F)	2018 N (F)	2021 N (F)	2018 N (F)	2021 N (F)	2018 N (F)	2021 N (F)	2018 N (F)	2021 N (F)	2018 N (F)	2021 N (F)
*Resource type*												
International guidelines	4 (23%)	7 (25%)	6 (35%)	6 (24%)	3 (17%)	7 (28%)	3 (17%)	3 (12%)	4 (23%)	4 (16%)	0	2 (8%)
National guidelines	5 (29%)	6 (35%)	9 (53%)	10 (40%)	5 (29%)	8 (32%)	4 (23%)	4 (16%)	4 (23%)	4 (16%)	0	2 (8%)
Medical Association Guidelines	0	0	0	0	0	0	0	0	0	0	0	0
Regional guidelines	0	0	1 (6%)	1 (4%)	0	1 (4%)	0	0	1 (6%)	1 (4%)	0	0
Institution guidelines	0	0	0	0	0	0	0	0	0	0	0	0
Meta analyses and systematic reviews	0	1 (4%)	0	1 (4%)	0	1 (4%)	0	0	0	0	0	0
Randomized controlled trials	0	0	1 (6%)	1 (4%)	0	1 (4%)	1 (6%)	0	1 (6%)	1 (4%)	0	0
**Other type of studies	0	0	0	0	0	0	0	0	0	0	0	0
Valid websites for institutions in that field	3 (17%)	2 (8%)	2 (12%)	2 (8%)	3 (17%)	4 (16%)	1 (6%)	1 (4%)	2 (12%)	2 (8%)	2 (12%)	2 (8%)
Specialized websites under the supervision of specialists in the field	1 (6%)	1 (4%)	1 (6%)	2 (8%)	1 (6%)	2 (8%)	0	1 (4%)	2 (12%)	2 (8%)	1 (6%)	3 (12%)
***Other information resources	0	0	0	0	0	0	0	0	0	0	0	0

*N (F): N = Number, F = Frequency

**Other types of studies: Observational studies, Cohort studies, Case–control studies, Cross sectional surveys, Case reports

***Other information resources: Laboratory research, Books, Expert opinions, News and non-specialized website, Wikipedia, Weblogs

The results related to the content type and information sources used in the development of cafe Bazaar applications are shown in Table 3. In 2018, the content types of most Cafe Bazaar applications were "general information on disease" and "prevention", and the most frequently used source in these applications was "The mentioned source unrelated to HIV/AIDS", each in 32% (n = 8) of the applications. In 2021, the most common content type was “Prevention”, and the most commonly used source was “The mentioned source unrelated to HIV/AIDS”. In 2018, among the Cafe Bazaar applications, in one application, the source was a national guideline, and in another application, it was a book. In 2021 these applications did not exist. None of the Cafe Bazaar applications reported that they used the evidences of the higher level of the hierarchy of evidences, such as different types of guidelines.

**Table 3:** The results related to the content type and information sources used in the development of cafe Bazaar applications

Content type	General information on disease		Therapeutic		Prevention		Self-care and self-management		Pharmaceutical information		Signs and symptoms	
	2018 N (F)*	2021 N (F)	2018 N (F)	2021 N (F)	2018 N (F)	2021 N (F)	2018 N (F)	2021 N (F)	2018 N (F)	2021 N (F)	2018 N (F)	2021 N (F)
*Resource type*												
**Guidelines	1 (4%)	0	0	0	0	0	0	0	0	0	0	0
***Some type of studies	0	0	0	0	0	0	0	0	0	0	0	0
Valid websites for institutions in that field	0	1 (3%)	0	1 (3%)	0	1 (3%)	0	0	0	0	0	1 (3%)
Specialized websites under the supervision of specialists in the field	0	1 (3%)	0	1 (3%)	0	1 (3%)	0	0	0	0	0	1 (3%)
Laboratory research	0	0	0	0	0	0	0	0	0	0	0	0
Books	1 (4%)	0	0	0	0	0	0	0	0	0	0	0
Expert opinions	0	0	0	0	0	0	0	0	0	0	0	0
News and non-specialized website	3 (12%)	8 (27%)	1 (4%)	2 (7%)	1 (4%)	7 (24%)	1 (4%)	3 (10%)	1 (4%)	2 (7%)	2 (8%)	4 (14%)
Wikipedia	2 (8%)	5 (17%)	2 (8%)	6 (20%)	2 (8%)	5 (17%)	1 (4%)	2 (7%)	0	0	2 (8%)	2 (7%)
Weblogs	1 (4%)	2 (7%)	1 (4%)	1 (3%)	1 (4%)	2 (7%)	1 (4%)	3 (10%)	0	0	1 (3%)	2 (7%)
The mentioned source unrelated to HIV/AIDS	8 (32%)	14 (48%)	6 (24%)	12 (41%)	8 (32%)	16 (8%)	2 (8%)	2 (7%)	2 (8%)	1 (3%)	7 (28%)	11 (38%)

*N (F): N = Number, F = Frequency

**Guidelines: International guidelines, National guidelines, Medical Association Guidelines, Regional guidelines, Institution guidelines

***Some type of studies: Meta analyses, systematic reviews, randomized controlled trials, observational studies, cohort studies, case–control studies, cross sectional surveys, case reports

### Results of rating the content type of applications

In this section, we just reviewed the applications that had mentioned the information acquisition sources. The mean rating of all application content in 2018 was 2.38 (SD = 0.74), and in 2021 was 2.90 (SD = 1.35) out of 5. The mean content rating of Cafe Bazaar applications in 2018 was 2.10 (SD = 0.49) and in 2021 was 1.94 (SD = 0.29), and the mean content rating of Google Play store applications in 2018 was 2.50 (SD = 0.80) and in 2021 was 3.86 (SD = 1.18) out of 5.

In 2018, three (17%) out of the 17 Google Play store applications, including Aids drug database (HIV Drugs), EACS, and HIV in practice rated above 4 (good).

In 2021, two (8%) out of the 25 Google Play store applications rated 5 (Excellent), including “WHO HTS Info”, and “WHO HIV Tx‏”. Also, 11 (44%) applications were rated above 4 (good).

In 2018, of the 25 Cafe Bazaar applications, three (12%) rated 3 (acceptable), nine (36%) 2 (poor), and 13 (52%) 1 (inappropriate). Neither of the HIV/AIDS applications evaluated in this study from the Cafe Bazaar was given the score 5 out of 5 by both reviewers.

According to the rating results of 2021 with using the EBCRT-mHealth tool, The top three HIV/AIDS Cafe Bazaar applications are: Agahibakhshi AIDS (Score = 3.1), Rahnamye AIDS va Bimarihaye Amizeshi (Score = 3.0), Zang khatar AIDS (Score = 2.58). Also, the top three Google Play Store applications are: WHO HIV Tx‏ (Score = 5), WHO HTS Info (Score = 5), EACS‏ (Score = 4.9), and ClinicalInfo HIV/AIDS Guidelines‏ (Score = 4.9).

The results of rating the type of application content in 2018 and 2021 are shown in Table 4. In 2018, The highest rates of content type in Cafe Bazaar applications was related to the content "identifying health and social service centers" (2.63 ± 1.22), and the lowest related to "information on conferences and events" (1.33 ± 0.47). Applications in the Cafe Bazaar also lacked information on “management of alcohol and drug use", "abbreviations and dictionary", and "diagnostic aid testing". The highest rates of the content type in Google Play applications were related to "therapeutic information" (3.0 ± 1.5) also in 2021 were (4.38 ± 1.08) the lowest was related to “management of alcohol and drug use" (1.27 ± 0.62), in 2021 were lack of information.

**Table 4:** The results of rating the type of applications content

Row	Rating the content type of Google Play store apps	Mean (SD) in 2018	Mean (SD) in 2021	Rating the content type of Cafe Bazaar apps	Mean (SD) in 2018	Mean (SD) in 2021
1	Therapeutic information	3.0 (1.5)	4.38 (1.08)	Identifying health and social service centers	2.63 (1.22)	2.69 (0.72)
2	Pharmaceutical information	2.92 (1.39)	4.16 (1.40)	General information on disease	2.48 (0.87)	2.01 (0.52)
3	Information on conferences and events	2.89 (1.2)	Lack of information	Prevention information	2.28 (0.87)	1.97 (0.54)
4	Identifying health and social service centers	2.83 (1.29)	3.70 (1.45)	Recent studies	2.18 (1.03)	Lack of information
5	General information on disease	2.82 (1.35)	4.26 (1.32)	Physical activity and fitness	2.13 (1.27)	1.60 (0.01)
6	Prevention information	2.68 (1.32)	4.03 (1.07)	therapeutic information	2.09 (0.82)	1.90 (0.56)
7	Self-care and self-management	2.34 (1.26)	3.90 (1.22)	Pharmaceutical information	2.07 (0.93)	1.92 (0.55))
8	Diagnostic aid testing	2.28 (1.57)	2.50 (0.71)	Signs and symptoms	2.02 (0.83)	2.08 (0.54)
9	Abbreviations and dictionary	2.24 (1.48)	3.79 (1.58)	Improving mood and emotion	2.0 (0.93)	Lack of information
10	Signs and symptoms	2.21 (1.32)	3.98 (1.20)	Self-care and self-management	1.93 (1.14)	1.93 (0.92)
11	Health tips	2.14 (1.21)	2.51 (1.27)	Health tips	1.88 (0.78)	1.93 (0.49)
12	Recent studies	2.08 (1.30)	3.70 (1.85)	Nutrition and diet	1.80 (1.25)	1.75 (0.35)
13	Improving mood and emotion	2.07 (1.13)	2.69 (1.27)	Information on conferences and events	1.33 (0.47)	Lack of information
14	Physical activity and fitness	1.42 (0.64)	4.05 (1.39)	Management of alcohol and drug use	Lack of information	Lack of information
15	Nutrition and diet	1.40 (0.76)	3.78 (1.62)	Abbreviations and dictionary	Lack of information	Lack of information
16	Management of alcohol and drug use	1.27 (0.62)	Lack of information	Diagnostic aid testing	Lack of information	Lack of information
	PEP and PrEP	2.90 (0.47)	3.90 (0.1)	PEP and PrEP	Lack of information	1.60 (0.01)

There was a significant relationship between the number of HIV/AIDS application downloads in the App Store and the EBCRT-mHealth tool score (*P*-value < 0.001, r = 0.599). In other words, with the increase of the number of downloads, the EBCRT-mHealth tool score increases too. Also, there was no significant relationship between app rates in the App Store with EBCRT-mHealth tool score (*P*-value = 0.146). The agreement between the two reviewers for the total score of the EBCRT-mHealth tool was ICC = 0.78 (CI95% = 0.67–0.86).

## Discussion

The objective of this study was to evaluate the content of HIV/AIDS-related mobile applications and the extent to which evidence-based knowledge was applied to their content. The results showed that the content of HIV/AIDS-related mobile applications available in Iran in 2018 was rated as "poor", and the content of few applications was "acceptable". Therefore, it is not easy to trust the content of most applications available in Iran, especially Cafe Bazaar applications.

In 2021, after almost two years, the Google Play Store HIV/AIDS mobile applications score increased from "poor" to "acceptable". But the score of Cafe Bazaar HIV/AIDS applications decreased from "poor" to "inappropriate". One of the reasons may be a large number of guidelines available in English compared to Persian. Another reason may be the policies for reviewing these applications and researching on the Google Play Store applications [39–41]. In Cafe Bazaar, there are no mobile application review policies, and very few studies have been done on these applications [42]. Mentioning the content acquisition source in mobile applications can help users assess content validity; also, users can refer to that source for more information [43, 44]. Given the important role of evidence-based education in the past few decades in transforming HIV/AIDS from a rapidly fatal disease to a chronic disease [5, 12, 13], using inappropriate and inaccurate information in these applications' content may adversely affect the treatment process.

The results also showed that the sources used in the Cafe Bazaar applications were at the lower of the evidence pyramid, and the sources used in the Google Play applications were at higher levels of the evidence pyramid. There was less than one score difference in 2018 and almost two score differences in 2021 between the total content rate of the Google Play Store and the Cafe Bazaar applications. A specific type of content in some Google Play store applications received a score of higher than 4, but due to the presence of other poor content with a low score, the overall rate of the application was low. The score of the content of most Cafe Bazaar applications was low. The source of information acquisition in most HIV/AIDS-related applications in Cafe Bazaar was unrelated. A review of the sources mentioned in the applications also showed that the application content was not found in the referenced sources. The results of the study by Robustillo Cortés et al. [26] showed that the quality of educational applications for HIV/AIDS positive patients is poor.

The organizational affiliation of more than half of the HIV/AIDS-related applications was unknown. The results of the study by Rosa et al. [25] also showed that more than half of the applications are not supported by health care organizations.

Checking the logo and title of the reviewed application revealed that only almost half of the applications did not appear to be on the HIV/AIDS topic. To prevent the sense of stigma in people that use these apps, and in order to preserve the confidentiality of these patients, it is suggested that uncustomary logos and names be used in the apps developed for these kinds of patients.

Mobile applications can be evaluated from different aspects and methods [18]. In the previously developed tools, such as Mobile Application Rating Scale (MARS) [19], Health On the Net (HON) [20], and DISCERN Instrument [21], along with other aspects, have questions about the evidence-based medicine and rated the evidence of the applications subjectively, without providing the basis for rating the evidences. In this study, we proposed an evidence-based pyramid that could be the basis for rating the evidence of the mobile applications. Also, we rated the applications according to this tool.

The most important advantage of using the content rating method developed in this study is the possibility of comparing applications based on content ratings using the hierarchy of evidence. Using the five-point ratings to rate the content of the applications enables the comparison of the results with the rating based on other tools [19, 21] that used the five-point rating method, as well as the five-star rating methods. Currently, users select a mobile application based on its popularity and the number of downloads, regardless of the quality of its information and content [45]. The evaluation method used in this study can be used to rate the content of mobile applications, along with the star rating method used by App Stores. We used this method to rate the content of HIV/AIDS-related applications; future studies can use this method to rate the content of other mHealth applications.

## Limitations

The present study had two limitations. First, this study was performed in Iran. Since all IOS and some Android applications are not available in Iran due to sanctions and regional restrictions of Google Play, the results may not be generalizable to other HIV/AIDS-related applications available in other countries. The researcher tried to access IOS mobile applications but was unable to access them in Iranian territory. The results of a previous study [27] showed that more than 60 percent of the HIV/AIDS mobile applications are available on both platforms. Also, another study [41] that evaluated 11 HIV pre-exposure prophylaxis mobile applications showed that all of these mobile applications are available in Android. Nevertheless, in terms of the number of HIV/AIDS-related applications, this study included the highest number of evaluated applications compared to previous studies [24–27]. Second, since it is not possible to pay for the Google Play Store in Iran, only the free applications were evaluated in this study. According to our searches in Google Play Store, all HIV/AIDS available mobile applications in Iran are free of charge. None of these applications have been excluded from the study due to impossible payment. The paid apps of Cafe Bazaar also entered to the study and were evaluated. A previous study [27] also reported that all HIV/AIDS-related applications of Google Play and iTunes were available free of charge.

In this study, the content evaluation was done based on the content of the applications at the time of downloading. Given that it is possible to update the applications in stores, we recommend developers to cite the source of the content in future updates of their applications and use reliable and evidence-based sources for developing content in order to increase the content rating of these applications.

## Conclusion

This study developed the EBCRT-mHealth tool and rated the HIV/AIDS mobile applications based on the evidence used in that mobile application content in 2018 and 2021. The results of this study showed, after almost two years, the rating of the content of HIV/AIDS-related applications available in Iran that existed in Cafe Bazaar decreased from "poor" to "inappropriate". Also, the Google Play Store applications' content score increased from "poor" to "good". It is critical to ensure the credibility of the sources used in developing their content and removing applications with inappropriate and unreliable content from the App Stores. Mobile health application developers can use the hierarchy of evidence pyramid developed in this study to ensure and demonstrate that they are using the highest quality information. Also, they can use this tool to evaluate their applications before sharing them in App Stores. The mobile applications researchers can use this tool to evaluate the applications in terms of evidence-based content and trustworthiness.

## Supplementary Information

**Additional file 1**. The EBCRT-mHealth tool.

## Data Availability

Not applicable.

## Abbreviations

EBCRT-mHealth: Evidence-based content rating tool of mobile health applications
MARS: Mobile apps rating scale
HIV: Human immunodeficiency virus
AIDS: Acquired immune deficiency syndrome
ICC: Internal correlation coefficient
SD: Standard deviation
WHO: World Health Organization
HON: Health on the net
CI: Confidence interval
