# Supplementary material for: Evaluation of HIV/AIDS-related mobile health applications content using an evidence-based content rating tool
Source: BMC Med Inform Decis Mak. 2021 Apr 24;21:135. doi: 10.1186/s12911-021-01498-7 (PMC8067376; doi:10.1186/s12911-021-01498-7)
Supplement: Supplementary file 1 — Additional file 1. The EBCRT-mHealth tool. [file 12911_2021_1498_MOESM1_ESM.docx]

**EBCRT-mHealth (Evidence-Based Content Rating Tool of Mobile Health Applications) tool**

**Section 1: Apps Information**

**App Name**: _________________________________________________

**Developer**: __________________________________________________

**Rate of the Apps in the store**: __________________________________

**Number of reviewer in the store**: _______________________________

**Version**: ______________________________ **Last update**: ____________________

**Platform**: IOS Android

**Store name**: Cafe Bazaar Google Play Store Apple App Store

**Affiliations**: Unknown Commercial Government NGO Academic

**Brief description**: ________________________

**Section 2: The type of content and resources**

Each of the following types of resources and types of content is used in mobile apps; mark that in the following table.

| R | Content type  Resource type | General information about condition | Therapeutic | Prevention | Self-care and self-management | Pharmaceutical information | Signs and symptoms | Others: like PEP and PrEP |
| --- | --- | --- | --- | --- | --- | --- | --- | --- |
| 1 | International guidelines |  |  |  |  |  |  |  |
| 2 | National guidelines |  |  |  |  |  |  |  |
| 3 | Medical Association Guidelines |  |  |  |  |  |  |  |
| 4 | Regional Guidelines |  |  |  |  |  |  |  |
| 5 | Institution guidelines |  |  |  |  |  |  |  |
| 6 | Meta analyses and Systematic reviews |  |  |  |  |  |  |  |
| 7 | Randomized controlled trials |  |  |  |  |  |  |  |
| 8 | Observational studies |  |  |  |  |  |  |  |
| 9 | Cohort studies |  |  |  |  |  |  |  |
| 10 | Case-control studies |  |  |  |  |  |  |  |
| 11 | Cross sectional surveys |  |  |  |  |  |  |  |
| 12 | Case reports |  |  |  |  |  |  |  |
| 13 | Valid websites of institutions in that field |  |  |  |  |  |  |  |
| 14 | Specialized websites under the supervision of specialists in the field |  |  |  |  |  |  |  |
| 15 | Laboratory research |  |  |  |  |  |  |  |
| 16 | Books |  |  |  |  |  |  |  |
| 17 | Expert opinions  (For example, noted according to the doctor x opinion ...) |  |  |  |  |  |  |  |
| 18 | News and non-specialized website |  |  |  |  |  |  |  |
| 19 | Wikipedia |  |  |  |  |  |  |  |
| 20 | Weblogs |  |  |  |  |  |  |  |
| 21 | The mentioned source unrelated to HIV/AIDS (We can use that when the mentioned source is not related to the subject) |  |  |  |  |  |  |  |
| 22 | Other: |  |  |  |  |  |  |  |

**Section 3: Ranking the content type of applications**

Based on this tool, if a type of content was not available in the application, a zero was assigned by the reviewers. For the available content, a score of 1 to 5 (inappropriate to excellent) was assigned to the mobile application.

The reviewers checked whether the content of the applications was consistent with the content of the source used in the development of the applications. To determine the source of information, all items that indicate the source of the content of the application, including links and hyperlinks, the mentioned sources at the end of the content, and the list of sources mentioned separately in the reference section inside the application should be considered. If a mobile application's content is based on a specific source, like a national guideline or several national guidelines are available in that mobile application. We assigned the national guideline score (score = 4.8) to that mobile application. To verify an application be a guideline or not to be, we checked the website registered in the Google Play Store, search on the internet, and details inside of that application.

If the application used several sources, like a reference list. We calculated the sum of all the sources' scores, and then the average score was considered. No score was assigned to the application if its content did not match the content of a related source. If the source was not available to determine the consistency and accuracy of the content, the application did not receive a score. The mean should be used to calculate each applications' average score and the total score of the applications.

| Row | Content type | Score 0 to 5  0: Content not available in the application  1=Inadequate, 2=Poor, 3=Acceptable, 4=Good, 5= Excellent |
| --- | --- | --- |
| 1 | General information about condition |  |
| 2 | Pharmaceutical information |  |
| 3 | Information on conferences and events |  |
| 4 | Identifying health and social service centers |  |
| 5 | Therapeutic information |  |
| 6 | Prevention information |  |
| 7 | Self-care and self-management |  |
| 8 | Diagnostic aid testing |  |
| 9 | Abbreviations and dictionary |  |
| 10 | Signs and symptoms |  |
| 11 | Health tips |  |
| 12 | Recent studies |  |
| 13 | Improving mood and emotion |  |
| 14 | Physical activity and fitness |  |
| 15 | Nutrition and diet |  |
| 16 | Management of alcohol and drug use |  |
|  | PEP and PrEP |  |
| 17 | Other |  |
